# Report of the Institution Established at Nottingham, in November, 1805, with the View of Exterminating the Contagion of Small-Pox, by Promoting a General Vaccine Inoculation, and More Particularly by Offering the Advantages of That Valuable Discovery without Expence, to Such of the Poorer Orders of the Community as Were Disposed to Accept Them

**Published:** 1806-08

**Authors:** James Clarke

**Affiliations:** Physician to the Institution. Nottingham


### *To the Editors of the Medical and Phyfical Journal.*

Gentlemen,

PERMIT me to present you with the first half-yearly Report of the Nottingham Vaccine Institution; to which I have subjoined a few observations.

I am, &c.

JAMES CLARKE,

Physician to the Institution.

*Nottingham, July* 9, 1806.

---

*Report of the Institution established at Nottingham, in November*, 1805, *with the view of exterminating the contagion of small-pox, by promoting a general vaccine inoculation, and more particularly by offering the advantages of that valuable discovery without expence, to such of the poorer orders of the community as were disposed to accept them.*

THE directors of this Institution have now arrived at the most agreeable part of their labours, that of reporting to the public their proceedings and success in the trust committed to them.

They have considered the duty imposed upon them as twofold.

1st, That of making an offer of the benefits of this practice to the poor, in such manner as would put them to the least inconvenience, and seemed the most likely to secure their acceptance of it, by removing those prejudices which must for a time obtain against a discovery, however valuable, that is yet new.

2d, That of establishing such rules for the conduct of the practical part of this undertaking, as, by enabling the Institution

Institution to exhibit a series of regularly recorded and conclusive facts, may best contribute to the improvement of a practice, which, though hitherto greatly successful, cannot be supposed to have arrived at that degree of perfection of which it is susceptible.

Guided by these views, the directors thought it best to commit the executive part of their undertaking to one surgeon, who engaged to devote a competent portion of his time to that object exclusively; to visit the different quarters of the town in rotation, for the purpose of inviting such of the inhabitants as were judged proper objects of a charitable establishment, to partake at their own houses of the benefits it held out: and lastly, to keep a regular record in a form prescribed by the Institution, of each case, exhibiting its appearance and progress at four different periods of the disease, when the patient was visited for that purpose. All this was to be transacted under the superintendence of a physician, who very liberally offered his services to the Institution with that view.

How far the plan of this institution has been calculated to secure the objects the directors have so much at heart; and how far the practical part of it has been conducted with skill, diligence and discrimination, the public will be enabled to judge, by a little attention to the leading facts now laid before them, which are meant to present an outline of the history of the Institution, for the six months that have elapsed since it began to act.

From the commencement of that period, viz. the 11th of Dec. 1805 to the 10th of June 1806 inclusive, there have been inoculated for the cow-pock, in all 1175, of whom 967 are regarded as complete and satisfactory cases; 98 are distinguished in the Register as unsatisfactory, because in these cases the pock having been broken by accident or scratching, its natural progress was so far altered or interrupted, as to render the protecting power of the disease from future small-pox somewhat doubtful; of course, the Institution does not charge itself with any responsibility in respect to these cases; the patients themselves, or their parents, having rejected a second inoculation, the only means of placing the matter beyond the reach of doubt.

A third set of cases, 69 in number, after being regularly inoculated and entered in the Register; the sequel is necessarily left blank, because the matter having failed to take effect at the first operation, no opportunity was afforded for a second, in consequence of a refusal of the

 patients patients or their parents; a change of their place of abode, or some similar reason.

The remaining 41 cases are under vaccination.

Every person who will take the trouble to reflect on this subject, will perceive how deeply the interests of this discovery, and the improvement of its practice, are concerned in a due distinction of the cases into their different classes of complete, doubtful, and imperfect.

It will be more difficult to convince parents and patients of the great utility of a second inoculation called the Test, a few days after the first, as the easiest, and in many cases, the only means of removing all uncertainty respecting the event; and by that means, reducing the class of doubtful or unsatisfactory cases to a very few indeed.

Parents are easily satisfied when the common appearances present themselves, whether the pock be broken or remain entire; but experience has proved, that in such cases, no reliance ought to be placed but on a second inoculation or test.

No application ought to be made to the broken pock, but by the positive direction of the vaccinating surgeon.

The directors feel a peculiar satisfaction in being able, within six months, to present the result of so extensive a practice to the public, without having a single instance of death to charge upon it; it is admitted, that one child of three months old, and of a weakly constitution, was on the 9th day after vaccination, taken with vomiting followed by convulsions, which put a period to its existence; but it ought to be remarked, that the inoculated part made no progress after the sixth day, and that this is usually observed to happen when any new disease arises; add to this, that the parents themselves were satisfied, that the cow-pock had no concern in producing their child's disease.

Much has been said and written respecting the frequency of foul eruptions, as a consequence of cow-pock inoculation; and this, in fact, is an objection that has been urged against the practice in this town and neighbourhood.

If eruptions be a frequent consequence in other parts, we have been peculiarly fortunate in not being able to report more than six instances, where eruptions have appeared within a month after the completion of the process, in nearly a thousand cases of vaccination; where they did occur, the eruptions were of a kind incident to children from from various causes, and speedily yielded to very simple treatment.

No other accident or inconvenience has been found to follow this disorder.

No instance of small-pox has occurred to any patient that has been vaccinated under the superintendence of the institution; and should any suspicion of this kind arise at any future period, it is earnestly requested, that a written communication to that purpose, may be sent without delay to any one of the directors, to Dr. Clarke, or Mr. Calton, at their houses, that the case, whatever it may be, shall undergo a fair and full investigation before it be too late to ascertain its nature.

The next observation we have to make, has a peculiar claim to the attention of the public, as affecting the main question at issue, *the protecting power of cow-pock from the contagion of small-pox.*

The small-pox was not known to be in the town at the time the practice of public vaccination was entered upon, but in a few weeks afterwards, the child of a traveller was brought to a lodging house in Charlotte Square, affected with the confluent small-pox, where others were exposed to the infection; the vaccinating surgeon, in compliance with his instructions on that subject, directed his whole attention to that neighbourhood, and by obtaining leave to inoculate the greater number of the poor thus exposed to the contagion, the natural small-pox happily ceased.

In the course of the two following months, the infection appeared in three other parts of the town, but was arrested in its fatal progress by the same means: we say *fatal progress,* because, out of seven instances of casual small-pox that came to our knowledge, two of the patients died, although medical aid was afforded them.

In one person who, although under the influence of small-pox contagion, had submitted to vaccine inoculation before the small-pox appeared, both diseases took place, but the small-pox was so mild that the patient recovered speedily, and with little inconvenience. From this, as a solitary case, no inference could be taken, but that it is found to concur with the experience of the most extensive vaccinators, who assert, that wherever the two diseases meet, the small-pox has generally proved of a mild sort. No greater argument can be offered to encourage vaccine inoculation, during an exposure to small-pox contagion, if the eruption has not actually appeared.

It is proper to remark, that the register makes it appear, that out of the number inoculated, 507 exceed the age of two years, a circumstance which is brought in proof of the indifference of parents to this practice, although they could have obtained it without expence, either at the general hospital, or by the liberal offer of many of the surgeons of this place: it follows, that the advantages of so general a cow-pock inoculation, are due to the plan adopted by the Institution; with all its advantages, a considerable number, that were proper objects, have rejected the offer from religious scruples. We have, however, reason to congratulate the public, that vaccination has in this town been carried to an extent in six months, which does not appear to have been equalled in any town of considerably greater population, the report of whose public practice for the last year has reached us. This will appear by a comparison of the report of the vaccine establishments in Dublin, Edinburgh, Bath, and Newcastle, some of them of double, or even nearly triple our population, with the present report for Nottingham.

| | | Inoculated. |
|---|---|---|
| Dublin, 2d Report for 12 Months | - | No. 1032 |
| Edinburgh, ditto ditto | - - | 1658 |
| Bath, 1st Report for City, ditto | - - | 354 |
| Newcastle upon Tyne, ditto | - - | 1708 |
| Nottingham, 1st Report for 6 Months | - | 1175 |

It remains for the public to judge, how far a charitable establishment for cow-pock inoculation in this place, has been attended with success, and how far it deserves future support; it is obvious, that this can only be done at a regular annual, and we think a moderate expence. Hitherto, the benefits may be regarded as confined to those individuals who have accepted them; the advantage to the community can only result from a permanent plan of early inoculation for cow-pock, such as may prove sufficient to prevent, or if it does appear, greatly to confine the fatal influence of the contagion of small-pox.

(Signed) G. COLDHAM, Secretary.

---

Independent of the practice of vaccination under the immediate direction of the Institution, as faithfully recorded in this report, some idea may be formed of its indirect extension, and the respect in which it is held in the vicinity of Nottingham, by the repeated applications for vaccine ichor, seventy-six separate supplies of which have been already gratuitously furnished.

The

The object of the above Report is to present to the public, a clear and candid statement of the practice of vaccination by this Institution, for the last six months. Professional men may not feel quite satisfied with the explanation given of the doubtful cases, after reading the assertion of Dr. Walker, in page 256, 545, Vol. 15th of the Medical and Physical Journal, where, he says, the induration or characteristic hardness surrounding the inoculated part about the tenth day, is the sure and invariable diagnostic mark of the true vaccine disease; but it is necessary at the same time to remember, that in page 431 of the same volume, we promised to put this opinion to the test of *experiment.*

Since which time, thirty-five cases of ruptured *pustule*, with *well marked induration* and erythema, have been re-inoculated.

Out of this number, three passed through the regular stages of vesicle, pustule, with distinct *induration* and erythema, scab of perfect form and colour. This is no hypothesis, but an undeniable statement, which the register of the Institution proves. Were *these* cases secured, had they been exposed to the variolous contagion before the last inoculation? We wish to put it as much as possible out of the power of any one to mistake our meaning. We say then, that *these three cases* had from the first inoculation a *proper degree of induration* and erythema on the tenth day; and the same result took place, and at the same time, by the last inoculation, three or four months after the first; but the scabs being preserved on the arm, presented the characteristic appearance so ably described by Jenner.

It gives us then, additional satisfaction to repeat, that no cases are by this Institution considered as perfect, that do not present the characteristic scab. But when the pustule is ruptured, re-inoculation is advised; if rejected, we do not consider ourselves responsible for the result.

The register presents one case, in which there was not any *induration* but a perfect scab; from which it appears that the characteristic scab is not a necessary result of the *induration*. These facts, which to us appear important, may be also thought deserving the attention of practical inquirers; their conclusions, however different, shall be appreciated with liberality and candour.

I shall beg permission to say a few words in answer to some of your correspondents.

In page 308, Mr. Creaser, and in page 319 of vol. 15th, Mr.

Mr. Ring, make some observations on the term *scorbutic*, which was employed in my letter addressed to the Royal Jennerian Society (for the style of which an explanation, has already been made, page 246, vol. 15th).

In answer to them, I shall explain what must be understood by the term *scorbutic*. It was used to express a constitution debilitated from bad food and foul air, with a roughness of the cuticle. I confess it was a vague term, but one that is well understood by medical men, although not nosologically accurate.

Mr. Ring, in page 320, vol. 15, comments on my remark respecting the pock being suspended during the presence of another disease in the constitution; it was not the suspension that was thought remarkable, but the peculiar appearance of the pock, where active disease was present; an appearance which I have not seen noticed in any other communication.

Dr. Walker, in page 544, vol. 15th, censures Mr. Ring "with *other labourers* in the vineyard," and calls upon him for a decided test of the vaccine disease.

I am not to be deterred by this criticism on practical remarks from saying, that we have not any proof, that the circumscribed characteristic hardness is a certain and invariable test of the vaccine disease. If practical observation enable a man to contradict an assertion, which, if implicitly acted upon, might be injurious, he certainly does not deserve rebuke, but the grateful acknowledgement of the liberal and enlightened.

Nor is it by any means necessary, that the person who point out the fallacy of an opinion, should be obliged hastily to advance one, probably equally fallacious, to be refuted in its turn.

At present I know *no other means* of security in cases of ruptured pustule, but repetition of inoculation.

In all cases where it may be necessary to repeat the inoculation, a vesicle should arise, which, if the subject have from the prior inoculation been completely vaccinated, will desquamate between the fifth and eighth day.

---

